# Migraine and tension-type headache in Germany. Prevalence and disease severity from the BURDEN 2020 Burden of Disease Study

**DOI:** 10.25646/6990.2

**Published:** 2020-09-09

**Authors:** Michael Porst, Annelene Wengler, Janko Leddin, Hannelore Neuhauser, Zaza Katsarava, Elena von der Lippe, Aline Anton, Thomas Ziese, Alexander Rommel

**Affiliations:** 1 Robert Koch Institute, Berlin Department of Epidemiology and Health Monitoring; 2 Evangelic Hospital Unna; 3 University of Duisburg-Essen, Department of Neurology; 4 EVEX Medical Corporation, Tbilisi, Georgia; 5 I.M. First State Medical University of Setchenov Moscow, Russia

**Keywords:** MIGRAINE, TENSION-TYPE HEADACHE, BURDEN OF DISEASE, MEDICATION, COMORBIDITIES

## Abstract

Headache disorders are widespread among women and men in Germany and are primarily associated with restrictions on quality of life. The two most common types of headache disorders are migraine and tension-type headache. In order to gain valid estimates of the prevalence of these conditions, a cross-sectional telephone-based survey was conducted among adults in Germany (N=5,009) between October 2019 and March 2020. The frequency, duration, the characteristics and comorbidities associated with headache were measured using the diagnostic criteria defined in the International Classification of Headache Disorders. 57.5% of women and 44.4% of men in Germany stated that they had had a headache in the last twelve months. 14.8% of women and 6.0% of men meet all of the diagnostic criteria for migraine. Tension-type headache affects 10.3% of women and 6.5% of men. Migraine and tension-type headache are predominantly found among people of working age and steadily decrease with age. Migraine is often accompanied by comorbidities such as depressive symptoms and anxiety disorders. People affected by headache disorders tend to receive very little professional medical care, with only a minority seeking treatment within a year. These results provide a comprehensive picture of the population-related impact of headache disorders and are used in the BURDEN 2020 study to quantify key indicators for burden of disease assessment.

## 1. Introduction

Headache is widespread and among the recurrent symptoms and disorders that many people experience [1]. Already in the telephone-based health survey GSTel 2004, conducted by the Robert Koch Institute (RKI), was reported that 66.6% of women and 53.0% of men in Germany have had a headache in the past twelve months [2]. Furthermore, the German Health Interview and Examination Survey for Children and Adolescents (KiGGS Wave 2, 2014–2017) found that 45.2% of 11- to 17-year-old girls and 28.7% of boys of the same age were affected by recurring headache during the last three months [3].

Several major epidemiological studies of the German population have provided estimates of the prevalence of tension-type headache and migraine – the two most common headache disorders. A migraine is characterised by a unilateral, pulsating or throbbing pain that patients describe as moderate to severe and which is usually aggravated by physical activity. Migraine may be accompanied by nausea, vomiting and photo- and phonophobia. If this condition is accompanied by additional restrictions to a person’s field of vision (flashing or lightning streaks) or language and speech disorders, it is referred to as migraine with aura. In some cases, one or more limbs may become numb during a migraine attack. In contrast, tension-type headache is characterised by a bilateral, pressing or tightening (non-pulsating) pain that patients describe as mild to moderate and which is not aggravated by physical activity. A tension-type headache may be accompanied by photo- and phonophobia, but not by both symptoms at the same time [4].

**Info box:**

**The burden of disease**

The Global Burden of Disease Study undertakes comprehensive calculations of the burden associated with disease throughout the world and, therefore, is an important informational system that can be used for international comparisons [15]. The study adds the figures for premature mortality (years of life lost, YLL) and healthy years of life lost due to illness (years lived with disability, YLD) to obtain a total measure known as DALY (disability-adjusted life years). Therefore, instead of simply comparing disease frequency, the study compares the impact that certain conditions have on population health, measured as loss of healthy lifetime. Since headache disorders are not a cause of death in the sense of the coding requirements defined by the World Health Organization (WHO), they are not included in YLL calculations. The proportion of the total burden of disease for headache disorders, therefore, is lower when expressed in DALY than when measured in YLD. However, migraine was still ranked ninth among all illnesses in 2017, and was responsible for 2.4% of DALY in Germany [54].

The frequency of headache disorders fluctuates due to the different types of sample and survey modes of the studies. Migraine frequency has been found to vary from between 15.6% and 23.9% among women and between 4.0% and 11.1% among men, whereas tension-type headache can vary from 14.5% and 31.7% among women and between 12.0% and 26.9% among men [1, 2, 5, 6]. In most healthcare statistics, the importance of general headache or of migraine and tension-type headache is not reported. As the majority of people affected by headache usually does not seek medical help and is normally unable to work for only a short period of time, it is difficult to use claims data or data on wage replacement benefits to estimate headache prevalence. Therefore, headache disorders are not among the 30 most common diagnoses made in general practices. Even in neurological outpatient practices, which provide professional care for people with these conditions, migraine (International Statistical Classification of Diseases and Related Health Problems, 10th Revision, ICD-10: G43) and other headache syndromes (ICD-10: G44) only represent 4.4% and 3.8% of all treated cases respectively [7, 8]. Accordingly, the health insurance provider Barmer Ersatzkasse reports that the proportion of their policy holders who are treated for headache disorder remains in the single-digit range [9, 10]. Headache (ICD-10: R51) and migraine (ICD-10: G43) also only account for 1.9% of cases of incapacity to work and 0.8% of days lost through incapacity to work among the insured people [10]. However, the costs associated with these conditions are higher once the indirect costs are taken into account. The indirect costs can be calculated using survey data and are caused by the reduction in performance and productivity of people who continue to work despite their condition. According to the European Union (EU) Eurolight study (2008–2009), indirect costs account for more than 90% of the total costs that are estimated to be associated with headache [11, 12].

The public health relevance of headache disorders is particularly apparent from the impact on quality of life that headache has on the people affected and those around them. Many people find that headache disorders severely restrict their ability to carry out normal daily activities. In addition to reduced performance at work, headache leads people to reduce their level of social contact and to avoid leisure activities [11, 13]. Even the fear of being affected by another headache episode can have a major impact on quality of life and promote further comorbidites such as depression, sleep disorders and other pains [13, 14].

The reduced quality of life due to illness-related restrictions is measured in the burden of disease assessment by calculating the loss of healthy life-years [15]. According to the Global Burden of Disease Study (Info box), in 2017 in Germany, migraine accounted for 5.1% of all years lived with disability (YLD) and ranked second among all illnesses. Migraine is associated with more severe restrictions on quality of life and, therefore, is more important than tension-type headache, which accounted for 0.7% of YLD and ranked 31st [16]. However, the reliability of the results from the Global Burden of Disease Study is limited due to difficulties with data availability [17–19]. As such, it is very important that headache disorders are integrated into national health surveys to improve the data pool available for calculating the burden of disease [20].

The study ‘BURDEN 2020 – Burden of disease in Germany at the national and regional level’ estimates the YLD for several important illnesses, including tension-type headache and migraine [21]. The aim of the study is to improve the data pool used for calculating the burden of disease in Germany and to adapt it to specific informational needs. Measuring the prevalence by using the criteria of the International Classification of Headache Disorders [4], and the operationalisation of severity levels for headache disorders according to the definition of the Global Burden of Disease Study [22], provides certain challenges. Relevant studies in Germany, were either conducted some time ago, cover only specific regions or do not provide all of the necessary information [1–3, 5, 6, 23–27]. Therefore, a population-based survey of pain disorders was conducted as part of the BURDEN 2020 framework in order to collect up-to-date data.

This article uses data from the study to provide an overview of headache disorders in Germany, and particularly of migraine and tension-type headache. It focuses on the frequency of headache disorders and their distribution by age and sex. It also outlines the severity of headache disorders in terms of their duration, intensity and frequency. The social determinants of headache disorders are also examined as part of a multivariate analysis. This article also analyses the utilisation of outpatient medical services and the use of medication by people with headache disorders, including the frequency of medication overuse.

## 2. Methodology

### 2.1 Data collection

A nationwide cross-sectional telephone survey of headache, back and neck pain was conducted in Germany between October 2019 and March 2020. The study targeted German-speaking residents of Germany aged 18 and older. It was developed by the RKI and carried out by USUMA GmbH, a market and social research institute. Implementation and quality assurance were performed based on a standardised concept [28]. Potential participants were contacted using a sample of landline and mobile phone numbers that had been randomly selected using the ADM (Working Group of German Market and Social Research Institutes) telephone sampling system [29]. The overall sample comprises 60% landline and 40% mobile phone numbers [29]. As every telephone number in Germany was eligible for the sample used by the study, it is representative of all households that are reachable by telephone in Germany. Landline telephone numbers were selected randomly using the Kish selection grid [28].

The study used a questionnaire that was based on those used by the RKI for other telephone-based health monitoring studies [28]. Validated instruments for measuring headache, back and neck pain were selected in advance and implemented as part of the questionnaire to ensure comparable results and to enable the identification of temporal trends [1, 30–34]. The study collected detailed information on the characteristics associated with individual pain disorders. Data were also gathered on general health and life satisfaction. Information on the medication use of people with headaches was also collected. On average, the interviews lasted 23 minutes. The questionnaire is available on request. The response rate, which was calculated using criteria drawn up by the AAPOR (American Association for Public Opinion Research), was 24.0% [35].

### 2.2 Measuring migraine and tension-type headache

For headache disorders, an instrument for migraine implemented in the RKI’s 2004 GSTel health survey has been further developed [5, 32]. The aim was to enable migraine and tension-type headache to be measured in line with the diagnostic criteria set out in the International Classification of Headache Disorders (ICHD – 3rd edition) [4], which is also used by the Global Burden of Disease Study [22]. The ICHD criteria can be summarised as four key indicators: (i) frequency, (ii) duration, and (iii) the characteristics of and (iv) comorbidities associated with headache attacks.

Table 1 provides an overview of how the indicators were built to identify people affected by migraine or tension-type headache in line with the ICHD criteria. In order to measure headache intensity, respondents were asked to classify their headache as mild, moderate or severe. Additionally, the mean frequency of headache attacks per month and the duration of attacks were also recorded. In accordance with the ICHD criteria, respondents were asked to estimate how long they would expect a headache to last if they did not use pain medication or if their medication was unhelpful. Participants are classified as suffering from migraine if they have had at least five attacks in their lifetime (A), lasting between four and 72 hours (B), meet at least two of four additional characteristics (C) and their headaches are accompanied by at least one of two specified accompanying symptoms (D). Cases that meet all four characteristics (A–D) are described as ‘definite migraine’, whereas those that meet three characteristics are categorised as ‘probable migraine’.

Participants are classified as suffering from tension-type headache if they have had at least ten attacks in their lifetime (A), lasting between 30 minutes and seven days (B), meet at least two out of four additional characteristics (C), and their headache is accompanied by the two specified accompanying symptoms (D). Cases that meet all four characteristics (A–D) are described as ‘definite tension-type headache’, whereas those that meet three characteristics are categorised as ‘probable tension-type headache’.

Migraine can be further categorised according to it´s clinical symptoms as migraine with or without aura (Table 1). Cases of (definite or probable) migraine that also includes restrictions on a person’s field of vision, weakness in their limbs or speech disorders are classified as migraine with aura (Table 1). Furthermore, (definite) migraine and (definite) tension-type headache can be subdivided into episodic headache (if they occur on less than fifteen days per month) and chronic headache (if they occur on fifteen or more days per month).

All participants who had had one or more headache in the past twelve months were given the opportunity to describe their headache. They were asked the following question: ‘Do you always have the same type of headache or do you have different types of headache?’ People who had different types of headache were asked two times to describe the frequency, duration, and characteristics, as well as the accompanying symptoms of their headaches. Each headache was then categorised either as a migraine or a tension-type headache in a way that the described headache could not meet all of the criteria for migraine and tension-type headache at the same time. Definite headache types were given priority over probable headache, which means that cases that met the characteristics for probable tension-type headache and definite migraine were classified as a migraine. Cases that met the characteristics for probable migraine and for definite tension-type headache were classified as a tension-type headache. Finally, a probable migraine was given priority over a probable tension-type headache.

**Figure 1:**
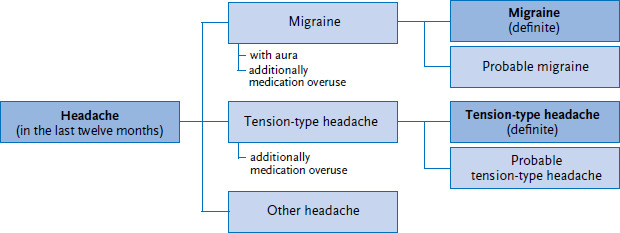
Schematic representation of headache based on the Global Burden of Disease study Source: Own diagram

The following analysis focuses on (Figure 1):

**►** People with headache in the last twelve months.
**►** People with (definite) migraine.
**►** People with (definite) tension-type headache.

In the following, the terms ‘migraine’ and ‘tension-type headache’ are used to refer to definite migraine and definite tension-type headache.

Headache due to medication overuse is a comparatively rare phenomenon with a prevalence of between 1% and 2% among adults [36, 37]. It can occur when a person takes a large amount of medication to treat acute pain, including pain caused by migraine and tension-type headache. In order to determine if participants are affected by this condition, respondents were asked to list up to five medications that they take as a prophylaxis or to treat acute headache and other pain conditions. The interviewers recorded the responses using an extensive list of medication (brand or trade names) that are used to treat headache and other pain disorders. Participants were also allowed to describe the active ingredients that their medication contained. Additional space was available to provide details about non-listed medications. The participants were also asked how long and how often they took the medication. The ICHD was used to categorise medication overuse among people who reported headaches on at least fifteen days a month and who took common pain relievers (e.g. paracetamol, ibuprofen, acetylsalicylic acid or mixed analgetics) on at least fifteen days a month or triptans, ergotamine or opioids on at least ten days a month. In both cases, the participants had to have taken the medication for more than three months.

### 2.3 Additional indicators

In addition to information on the frequency of certain types of headache in the population, further information was collected to better describe the respondents' headache disorders. This includes information about other diseases and conditions that the respondents reported. Studies have identified relevant associations between pain and mental disorders [13]. Depression and anxiety disorders are particularly relevant in this context. Therefore, data on depressive symptoms and anxiety disorders were recorded and this was done by collecting self-reported information on the four items set out in the Patient Health Questionnaire (PHQ-4). The symptoms reported were then assessed according to the frequency of their occurrence over the past two weeks. The criteria for depression and generalised anxiety disorder defined by the American Psychiatric Association’s Diagnostic and Statistical Manual of Mental Disorders (DSM-V) include two items for each of the diseases. A scale of zero to six was used in both cases; exceeding the threshold by at least three points was regarded as an indication of depressive symptoms or an anxiety disorder [38]. In addition, data on back and neck pain were collected using self-reported information on twelve-month prevalence, since these conditions can also be associated with migraine and tension-type headache [13]. A particular focus was placed on respondents who reported severe to very severe back or neck pain.

Furthermore, data were also collected on sociodemographic factors such as education, household composition and employment status. A distinction was made between unemployed people, people looking for work and employed or retired people. The 2011 International Standard Classification of Education (ISCED), which was drawn up by the United Nations Educational, Scientific and Cultural Organization (UNESCO), allows for international comparisons of educational levels and for classifying the highest achieved level of education in three groups (low, middle, high) according to EU standards [39]. Information on whether the respondent lived with a partner was also taken into account. Perceived levels of social support in a participant’s personal live were used as an auxiliary variable to measure possible support networks. Social support was measured using the Oslo 3 Social Support Scale, which is composed of three indicators [40]: (i) the number of close confidants who can be relied upon in the event of serious problems, (ii) the sense of concern that other people show the participant and (iii) the ease of gaining practical help from neighbours. This allowed to differentiate between ‘low support’, ‘medium support’ and ‘strong support’.

### 2.4 Statistical analysis

In order to ensure that the sample was representative for the population in Germany, it was adjusted for an individual’s probability of selection (design weighting) and for population structure (adjustment weighting). Design weighting was used to correct for the sample, where a method determining the selection probability in a telephone-based sample consisting of both landline and mobile phone numbers (dual frame design) was applied [29]. Adjustment weighting was carried out iteratively by sex, age, education and region to ensure that the distribution of these characteristics is similar to that found in the resident population in Germany aged 18 and older. Data from the Federal Statistical Office (population as of 31 December 2018) and the microcensus from 2017 served as a basis for this comparison [41, 42].

Statistical associations between various characteristics were also tested. The Pearson χ^2^ test for survey samples was used for nominal data [43]. With regard to purely metric values, differences in mean values between groups were determined using the t-test [44]. Multinomial logistic regression was also used to identify the social determinants of headache disorders [45–47]. The target variables were probable and definite migraine, tension-type headache and the remaining group of all other headache types. Participants with both migraine and tension-type headache were assigned to the migraine group. Participants without headaches served as the comparison group. A statistically significant difference between groups is assumed when the corresponding p-value is smaller than 0.05. All analyses were carried out using STATA (StataCorp LLC, Texas, US) version 15.1. All of the results presented in this article, including those with 95% confidence intervals (95% CI), are weighted and obtained using the survey procedures for complex samples.

## 3. Results

### 3.1 Sample

A total of 5,009 respondents (unweighted figures) participated in the study on headache, back and neck pain in Germany; 2,634 were women (52.6%) and 2,375 were men (47.4%). The distribution of the sample by age, sex and education shows that the study participants tended to be somewhat older and more educated than the population update of the Federal Statistical Office and the microcensus (Annex Table 1).

### 3.2 Frequency of headache disorders

**Figure 2:**
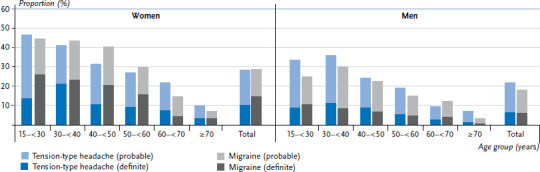
Prevalence of migraine and tension-type headache in adults by sex and age (n=2,634 women, n=2,375 men) Source: Study on headache, back and neck pain in Germany (2019/2020)

A total of 57.5% of women and 44.4% of men reported that they had at least one headache in the twelve months before the survey. 14.8% of women and 6.0% of men were affected by migraine that met all of the diagnostic criteria. 9.1% of those affected by migraine have chronic headache. 1.2% of participants have chronic migraine and 9.6% have episodic migraine. Many other respondents with recurrent headache only met three out of the four characteristics and, therefore, were classified as having probable migraine. If these figures are added to those for definite migraine, the proportion of people affected by migraine increases substantially to 28.4% of women and 18.0% of men. In addition, these people may also have symptoms of aura. Self-reported data show that 32.1% of women with definite migraine also meet the characteristics for migraine with aura, as do 42.9% of men. Women are more likely to experience migraine, but men report symptoms of aura more often than women. In addition, migraine is more common among younger women and men, and decreases steadily with age. Prevalence is highest among women aged between 18 and 29 years, and among men aged between 30 and 39 years (Figure 2).

10.3% of women and 6.5% of men meet all of the diagnostic criteria for tension-type headache. Symptoms of chronic tension-type headache are reported by 3.4% of those affected. Overall, 0.6% report chronic and 8.1% episodic tension-type headache. Again, if these figures are added to those for probable tension-type headache, 28.2% of women and 21.8% of men are affected by this condition. As is the case with migraine, tension-type headache is more common among people of working age and decreases steadily with age. Men have a lower prevalence of overall headache as well as for the two most common types, migraine and tension-type headache, than women. The differences identified between women and men remain stable within the age groups.

**Figure 3:**
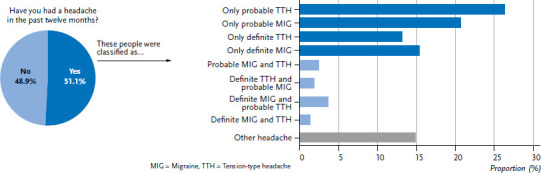
Distribution of headache in the past twelve months by headache type (N=5,009) Source: Study on headache, back and neck pain in Germany (2019/2020)

Figure 3 shows the distribution of participants suffering at least one headache over the past twelve months by diagnostic criteria. The graphs demonstrate that some respondents are affected by both migraine and tension-type headache. Overall, 51.1% of participants reported that they had had a headache in the past twelve months. Around one third of these respondents (35.0%) reported two different types of headache. 14.9% of respondents described headache that could not be clearly categorised either as a migraine or as a tension-type headache. 75.6% of respondents suffering from headache had either a migraine or tension-type headache. Approximately one in ten (9.5%) people suffering from headache showed symptoms of both migraine and tension-type headache (Figure 3).

**Figure 4:**
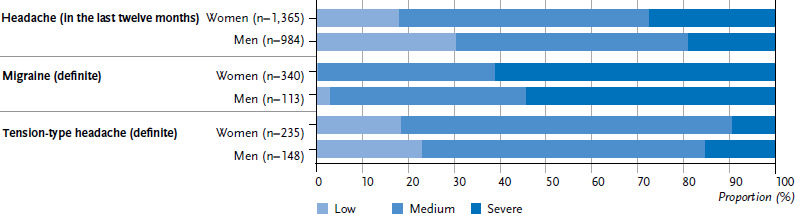
Intensity of headache, including (definite) migraine and (definite) tension-type headache Source: Study on headache, back and neck pain in Germany (2019/2020)

Among respondents suffering from headache, women are the most likely to report that they have had a moderate (54.4%) or severe (27.7%) headache in the past twelve months (Figure 4). Men are more likely than women to report a mild (30.2%) or moderate (50.6%) headache. This tendency also largely applies to respondents with migraine or tension-type headache. Women with migraine tend to describe their pain as severe (61.3%) or moderate (38.4%). This trend is particularly strong among women with chronic migraine, of whom, 79.3% describe their headache as severe. The proportion of men who have severe attacks is lower at 54.5%. However, similarly to women, 72.1% of men with chronic migraine describe their pain as severe. Respondents generally describe the pain associated with tension-type headache as moderate (women 72.0%, men 61.7%); in contrast to the results for migraine, this also applies to chronic tension-type headache.

On average, women are affected by overall headache on more days per month than men (Table 2). No significant sex-specific differences were found for tension-type headache. On average, men who are affected by migraine are affected slightly more often per month than women. However, this difference is not significant. The data for the twelve-month period show that, on average, headache lasts longer for women than men, and this also applies to migraine (Table 2). Both women and men with definite tension-type headache report that their headache lasts less than a day. No noteworthy differences with regard to the length of the headaches were found between respondents with chronic compared to those with episodic headache.

**Figure 5:**
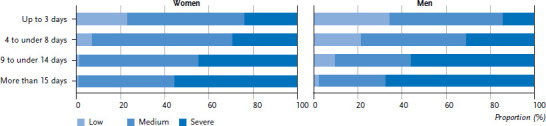
Intensity of headache over the past twelve months by frequency of attack per month (n=1,320 women, n=957 men) Source: Study on headache, back and neck pain in Germany (2019/2020)

An association was found for both women and men between the intensity and frequency of headache in the past twelve months, with intensity increasing with the frequency of headache attacks (Figure 5). 24.0% of women who have headache on less than three days a month describe them as severe, 55.7 % of women who have headache on more than fifteen days a month describe them as severe. A similar picture emerges among men: when they have a headache on less than three days a month, 14.4% describe them as severe, whereas, among those who have headaches on more than fifteen days a month, 67.6% describe them as severe.

### 3.3 Comorbidities associated with headache

As headache may be accompanied by certain physical illnesses and by higher levels of mental stress [13], this study examined the links between selected conditions and migraine and tension-type headache. Table 3 shows the proportion of participants with comorbidities within the groups of people with migraine, tension-type headache or without headache. The respondents without any headache during the last twelve months were used as the comparison group (sigificancy test).

Respondents with migraine and tension-type headache often report severe back pain (migraine 30.0%, tension-type headache 23.7%) or neck pain (migraine 31.4%, tension-type headache 19.2%) (Table 3). This is most evident when the results are compared with those for people without headache: only 12.6% of people without headache report severe back pain and only 6.7% describe severe neck pain. A high proportion of people with migraine also displayed depressive symptoms (24.9%) or an anxiety disorder (20.5%). In comparison, 15.3% of those without headache reported depressive symptoms and 8.7% showed symptoms of an anxiety disorder. Very few sex-specific differences were identified for the comorbidities considered in this study. Participants with chronic migraine reported severe neck pain (62.5%) and depressive symptoms (71.6%) particularly often. In contrast, chronic tension-type headache is not associated with a higher frequency of any of the comorbidities under consideration.

### 3.4 Outpatient medical treatment and the use of medication

Among participants who reported any headache in the twelve months before the survey, 22.0% of women and 17.1% of men stated that they had consulted a doctor about their condition. The results are similar for participants with tension-type headache: 22.1% of women and 17.0% of men consulted a doctor in the twelve months before the survey about their condition. However, people with migraine reported a higher rate of medical treatment: 40.6% of women affected by migraine and 38.5% of men with migraine sought medical treatment for their headache in the twelve months before the survey. Moreover, women and men who reported chronic migraine have received treatment significantly more often with 59.2% and 79.6%, respectively. This also applies to people with chronic tension-type headache (women 71.8%, men 24.0%).

Of all participants who reported any headache in the last twelve months, 82.5% of women and 67.0% of men stated that they take medication to treat acute pain. Among those with (definite) migraine, the numbers are significantly higher at 90.9% of women and 83.1% of men. Almost all respondents with chronic migraine take medication to treat acute headaches and other pain (women 97.0%, men 96.3%). Among those with (definite) tension-type headache, 92.8% of women and 75.5% of men take medication to treat acute pain. The proportions of women and men who take medication for chronic tension-type headache are lower than those who do so for episodic headache (women 73.4%, men 37.3% vs. women 93.9%, men 74.9%). A significantly lower proportion of people uses medication to prevent headache or other pain: 3.2% of all respondents with any headache in the last twelve months, 4.7% of those with migraine (chronic migraine 9.8%) and 1.6% of people with tension-type headache (chronic tension-type headache 4.2%) used medication as a prophylaxis. Only slight differences by sex were found.

Women not only take medication to treat acute headaches and other pain more frequently than men, but also do so more regularly. Among all respondents who reported any headache in the last twelve months, women take medication on 4.4 days a month on average, with men doing so on 2.8 days a month. In contrast, no significant differences were identified between women and men for (definite) migraine or (definite) tension-type headache. On average, respondents with migraine use medication to treat acute headaches and other pain on 5.8 days a month; participants with tension-type headache do so on 3.0 days a month. On average, medication is used to treat acute pain associated with chronic migraine on 14.7 days a month, and on 9.9 days a month for chronic tension-type headache. The most commonly used drugs are ibuprofen, paracetamol and acetylsalicylic acid (Table 4). Triptans, which are only used to treat migraine, play a greater role among people with (definite) migraine compared to all respondents with headache. Therefore, triptans are particularly taken by people with migraine and are ranked at place five with 7.3%. Since approximately one in ten people with headache showed symptoms of both migraine and tension-type headache, some respondents with definite tension-type headache also take triptans. Finally, no significant sexspecific differences were identified by type of medication.

It appears that in a small group of participants with chronic headache these headaches only occur or are exacerbated when medication overuse is also present. Specific therapeutic approaches, including the withdrawal of pain medication, may be indicated in these cases. Signs of headache caused by medication overuse are identifiable among 1.9% of people with migraine and 0.9% of people with tension-type headache. The prevalence among all adults is 0.9%. Furthermore, an additional 2.0% appear to take a higher than recommended dosage of medication for episodic headache.

### 3.5 Social determinants of headache

Multivariate analyses were carried out to investigate the possible social determinants of migraine and tension-type headache (definite: Table 5; probable and other headache types: results on request).

The results of the multivariate analyses demonstrate that women are almost 4.7 times more likely to have a definite migraine than men (Table 5). Women also are almost 2.7 more likely of having tension-type headache than men. With regard to age, the results for both conditions essentially confirm the findings from the descriptive analyses: the likelihood of having a migraine and tension-type headache steadily decreases with age (Table 5). No statistically significant associations between migraine or tension-type headache and education, unemployment or partnership were found. However, an association was found between definite migraine and social support: respondents with a high level of social support are less likely of suffering from migraine than respondents with a medium level of social support.

## 4. Discussion

57.5% of women and 44.4% of men in Germany report having had a headache at least once within the last year. However, the figures are lower when the clinical criteria for migraine and tension-type headache are applied. In this case, 14.8% of women and 6.0% of men were affected by definite migraine and 10.3% of women and 6.5% of men were affected by definite tension-type headache. The frequency of migraine and tension-type headache decreases significantly with age. Among all respondents with headache, women report mild headache less often and moderate or severe headache more often than men. The largest group of respondents who reported severe pain is women with migraine. Moreover, migraine is particularly frequently associated with comorbidities such as back or neck pain, depressive symptoms and anxiety disorders. In Germany, people with headache receive a low level of treatment, with 22.0% of women and 17.1% of men consulting a doctor within a year about their condition. People with migraine have a higher rate of treatment. Finally, 82.5% of women and 67.0% of men suffering from headache use medication to treat acute pain. The most common drugs taken are ibuprofen, paracetamol and acetylsalicylic acid.

When comparing these results to other findings, it is important to take into account the methodological differences in how studies identify migraine (or tension-type headache). Variation between studies can be explained by questionnaire design, the implementation of the diagnostic criteria using a wording understandable by non-specialist people, survey type, survey period and the approach taken in cases where an individual is affected by various types of headache [1]. The current study reports a smaller proportion of people who have had headache in the past twelve months compared to a telephone survey undertaken in 2004. The 2004 survey, which was also carried out by the RKI, found that 66.6% of women and 53.0% of men reported any headache in the last twelve months compared with 57.5% of women and 44.4% of men in the present survey [2]. The 2004 study also identified a somewhat higher proportion of respondents with (definite) migraine: 15.6% of women and 5.3% of men [2]. However, in addition to slight variations in how the two studies define migraine, the differences in prevalence could also be due to differences in the questionnaires (such as the order in which the questions were asked, or how the diagnostic criteria were integrated into the study). A study that compared headache prevalence in Germany between the 1990s and 2009 found little evidence of temporal trends [48].

There is only a small number of population-wide studies on headache disorders in Germany that have implemented the diagnostic criteria. Moreover, some of the studies that do so only cover specific regions [24] or certain age groups [3, 6, 25, 26, 27]. This limits their comparability with the results set out here. However, the headache study conducted by the German Migraine and Headache Society (DMKG study) combines the data from the Study of Health in Pomerania (SHIP), the Cooperative Health Research in the Augsburg Region (KORA) study and the Dortmund Health Study (DOGS) [5]. Nevertheless, it is important to note that each of these studies collected headache data differently. The DMKG study found that definite migraine affects 10.0% of women and 2.2% of men. Definite tension-type headache was identified among 19.7% of women and 17.2% of men. As such, the present study recorded a higher prevalence of migraine and a lower prevalence of tension-type headaches than the DMKG study. However, if the studies on which the DMKG overview report is based are considered separately, the prevalence estimated by the present study are actually similar for migraine compared to the DOGS and KORA studies, and consistently lower for tension-type headache [5].

The German Headache Consortium (GHC) Study, which provides results for specific regions in Germany (Essen, Münster and Sigmaringen), found that 19.1% of women and 7.1% of men suffer from (definite) migraine [6]. These values are slightly higher than the results presented in this study. The GHC study found that 3.8% of women and 3.4% of men are affected by (definite) tension-type headache. The results presented here are higher, at 10.3% for women and 6.5% for men. Both studies, the DMKG and GHC, differentiate between definite and probable migraine. However, the identified prevalence is drawn from different recall periods (six months in the case of the DMKG, and twelve months in the case of the GHC), the two studies refer to different regions and vary in the methods used to record migraine and tension-type headache [1]. In contrast, a population-wide survey from 2016 found that 7.2% of the population is affected by migraine and 12.4% by tension-type headache [27]. However, these results refer to the last six months and – as was the case with the DMKG study – the diagnostic criteria were not implemented. Instead, the participants were asked whether they had ever been medically diagnosed as having a headache (including migraine and tension-type headache).

Other findings presented here are comparable with the results of other studies. Other studies have provided similar estimates of the number of days that people are affected by headache per month. In a study conducted in the 1990s, participants with migraine described having a headache on 2.8 days per month, whereas those with tension-type headache reported headache on 2.9 days per month [49]. The DMKG study found that people with migraine have headache on average for four days a month [36]. Similarly, the present study estimated a mean of 4.1 days per month for migraine and 2.4 days per month for tension-type headache. A larger study of people with migraine in the United States also found a similar average duration of attack of around one day [22]. In addition, whereas the US study found that almost two-thirds of participants with migraine described the pain intensity as severe [49], in the present study, 59.4% of respondents with migraine described their pain as severe. The results presented here also describe important comorbidities related to migraine and tension-type headache: depressive symptoms, anxiety disorders and back and neck pain are the most important comorbidities. However, it must be noted that this is not an exhaustive list. There are many other notable comorbidities, such as fibromyalgia and sleep disorders, that may also show similarly strong associations with headache disorders.

An earlier population-wide survey of Germany estimated the use of medical care to be 41.6%, which is slightly higher than in the present study [2]. The present study found that 40.0% of people with migraine and 20.2% of people with tension-type headache coordinate their treatment with a doctor. Another population-wide study from 2016 found that almost one in two people with headache consult a doctor [50]. The overall rather low level of care and scarce attempts to seek help by people affected by headache disorders may be explained through their use of self-medication. It can therefore be assumed that most people with migraine and tension-type headache who use medication-based prophylaxis do not follow medical guidelines. The rare use of triptans to treat acute pain further suggests that these patients do not necessarily follow the recommendations set out by professional medical associations. As such, it would be important to discuss the ways in which impartial medical advice can be improved to encourage people with headache disorders to make informed decisions and to follow guideline-based care.

The risks associated with not seeking medical help and with self-medication include the development of chronic pain, and medication overuse [9–11]. Medication overuse can itself be associated with headache. The prevalence of medication overuse headache is estimated to be up to 2% [37, 51]; the DOGS, KORA and SHIP studies estimated the prevalence to be around 1% [36]. The current study found a prevalence of around 1% for people with headache disorders. It can be assumed that the frequency of medication overuse headache strongly depends on the type of survey and other study characteristics, which means that it is very difficult to provide a precise estimate of the prevalence of this type of headache.

The results presented here provide up-to-date and reliable information about the overall incidence of headache disorders in Germany. The sample design on which the results are based, the large number of participants (over 5,000) and the weighting used, ensure a high degree of representativeness. By translating the diagnostic criteria of the ICHD into language that is easy to understand, headache disorders such as migraine and tension-type headache can be distinguished from other types of headache. Furthermore, this study has demonstrated the feasibility of identifying different headache types even if the same respondent is affected by more than one form of the disorder. As the respondents don’t need to decide for one of the headache for which to report, a possible underreporting is not expected. However, integrating diagnostic criteria into studies can be challenging as the criteria can be translated in different ways, and respondents still need to provide information, for example, about the frequency of headache attacks that occurred over different study periods (their lifetime, a period of twelve months). However, recurring headache has a great impact on people’s lives, and it should therefore be possible for respondents to classify the type of pain that they experience. Nevertheless, it is impossible to rule out the risk that individual indicators such as migraine with aura or headache caused by medication overuse may either be over or underestimated. The same applies to chronicity, which this survey was unable to fully account for. However, the smooth implementation of the study in the field, the small number of missing values and the plausibility of its findings suggest that the respondents certainly understood the questionnaire.

As limitation can be noted that the study is affected by decreasing willingness of general participation in surveys. The study is also based on the assumption that everyone over the age of 18 years can be reached equally by telephone and that all possible landline connections and mobile phone numbers were included in the overall sample. The response rate was calculated using the American Association for Public Opinion Research’s criteria for ‘response rate 3’. In some cases, calculations that use other criteria may produce significantly higher response rates. However, the response rate reported here is comparable to that of studies conducted by the RKI and others [52]. Since the sample was adjusted to correct for specific characteristics, the data quality can be regarded as high. Nevertheless, it is unclear whether, for example, people suffering from chronic illness are adequately represented in the study and, if not, what impact this could have on the prevalence of headache disorders. Furthermore, estimates that are based on a small proportion of respondents, such as people with medication overuse or highly frequent headache attacks, are subject to a degree of uncertainty.

### 4.1 Outlook

Headache disorders are often very stressful and have a severe impact on the quality of life of the people who are affected and those around them. Since relatively few people with headache disorders seek medical treatment or receive wage replacement benefits, headache disorders are not particularly evident in claims data. However, studies that focus on the burden of disease and that quantify the loss of healthy lifetime due to illness in a population clearly demonstrate the public-health relevance of headache disorders.

The BURDEN 2020 study uses a population survey to quantify the prevalence and severity distribution of pain disorders as part of a national study of the burden of disease. This procedure has been proven to be effective in the case of migraine and tension-type headache. In order to measure prevalence, the criteria set out in the ICHD were used to define migraine and tension-type headache. This study was able to properly implement the approach used by the Global Burden of Disease Study. There is very little difference between the prevalence identified by the Global Burden of Disease Study [20] and the present study. The main difference between the study results is the lower prevalence of tension-type headache, which diminishes the burden associated with this condition in the national study’s calculations of YLD.

This article has not taken the next step of estimating severity distributions for migraine or tension-type headache. The Global Burden of Disease Study provides global (country-unspecific) estimates of severity distribution. In the case of many diseases, country-specific estimates deviate significantly from these figures [17, 19]. As such, different results can also be expected for Germany. In addition, the BURDEN 2020 study also provides estimates for Germany at the regional level. Claims data and older study data provide evidence of regional variation that should be further analysed [1, 2, 5]. Due to rather small case numbers in population based studies regional estimates can often only be made using small area estimation techniques. The RKI has already carried out preliminary work on this subject, which will be further developed using the example of migraine and tension-type headache for the purposes of disease burden calculations within the BURDEN 2020 study [53].

## Key statements

- 14.8% of women and 6.0% of men in Germany suffer from migraine; additionally, 13.7% of women and 12.0% of men have probable migraine.
- 10.3% of women and 6.5% of men in Germany are affected by tension-type headache; additionally, 18.0% of women and 15.3% of men have probable tension-type headache.
- Women from all age groups are more likely to experience migraine and tension-type headache than men.
- Migraines, in particular, are accompanied by a higher frequency of depressive symptoms and anxiety disorders.
- Tension-type headache and migraines occur most frequently among people of working age and decrease steadily with age.

## Figures and Tables

**Table 1:** Indicators to measure migraine and tension-type headache Source: Own table

	**Migraine (without aura)**	**Tension-type headache**
A. Frequency	At least five episodes over a person’s lifetime	At least ten episodes of headache occurring over a person’s lifetime
B. Duration of the attacks	4–72 hours (when untreated or unsuccessfully treated)	Lasting from 30 minutes to seven days (when untreated or unsuccessfully treated)
C. Characteristics	At least two of the following four characteristics: unilateral location Pulsating quality Moderate or severe pain intensity Aggravation by or causing avoidance of routine physical activity (e.g. walking or climbing stairs)	At least two of the following four characteristics: Bilateral location Pressing ot tightening (non-pulsating) quality Mild or moderate intensity Not aggravated by routine physical activity such as walking or climbing stairs
D. Comorbidities	During headache at least one of the following: Nausea and/or vomiting Photophobia or phonophobia	Both criteria are met: No nausea or vomiting No more than one of photophobia or phonophobia
**Additional indicator**	**Migraine (with aura)**	
	Criteria for Migraine (without Aura) are fulfilled In addition, the person fulfills at least one of the following criteria: Experiencing flimmer or flash in front of eyes, for at least 5 minutes Weakness, paralysis or numbness of limbs, or impaired speech	

**Table 2:** Frequency per month and duration for headache in general, (definite) migraine and (definite) tension-type headache Source: Study on headache, back and neck pain in Germany (2019/2020)

	Sex	Headache (last twelve months)			Migraine (definite)			Tension-type headache (definite)		
		AM	MD	STD	AM	MD	STD	AM	MD	STD
Days with a headache (in days per month)	Women	**3.2**	1.0	5.3	4.0	1.5	6.2	2.6	1.0	4.5
			(n=1,321)			(n=332)			(n=231)	
	Men	**2.4**	1.0	4.6	4.4	1.0	6.6	2.2	1.0	4.0
			(n=958)			(n=111)			(n=143)	
Duration of the headache (in days)	Women	1.0	0.1	2.1	**1.3**	1.0	1.1	0.6	0.1	1.1
			(n=1,289)			(n=340)			(n=235)	
	Men	0.8	0.1	2.4	**0.8**	1.0	0.7	0.6	0.1	0.8
			(n=936)			(n=113)			(n=148)	

Bold = A significant difference in the mean between women and men (p-value <0.05)

AM = mean, MD = median, STD = standard deviation

**Table 3:** The most common comorbidities Source: Study on headache, back and neck pain in Germany (2019/2020)

	Back pain	Neck pain	Depressive symptoms	Anxiety disorder
Migraine (definite) (n=449)	**30,0 %**	**31,4 %**	**24,9 %**	**20,5 %**
Tension-type headache (definite) (n=383)	**23,7 %**	**19,2 %**	12,6 %	10,2 %
No headache^1^ (n=2,632)	12,6 %	6,7 %	15,3 %	8,7 %

^1^ People answered ‘No’ to the question ‘Have you had a headache in the past twelve months?’

Bold = Significant difference (p-value <0.05) compared with participants without headache; comparison based on the Pearson χ^2^ test

**Table 4:** Use of medication to treat headache (percentage of all responses, multiple answers possible)^1^ Source: Study on headache, back and neck pain in Germany (2019/2020)

Medication	Headache (last twelve months)	Medication	Migraine (definite)	Medication	Tension-type headache (definite)
Ibuprofen	49.7%	Ibuprofen	46.2%	Ibuprofen	47.8%
Paracetamol	18.7%	Paracetamol	17.1%	Paracetamol	22.5%
Acetylsalicylic acid	15.0%	Acetylsalicylic acid	10.2%	Acetylsalicylic acid	14.9%
Metamizole sodium	6.0%	Metamizole sodium	9.3%	Metamizole sodium	4.0%
Triptan	2.8%	Triptan	7.3%	Diclofenac	2.6%
Diclofenac	2.3%	Diclofenac	2.4%	Triptan	2.4%
Naproxen	0.9%	Naproxen	1.2%	Celecoxib	1.0%
Tilidine	0.5%	Tramadol	0.7%	Naproxen	1.0%

Mentions	2,320	Mentions	578	Mentions	423
Respondents	2,349	Respondents	453	Respondents	383

^1^ This table only provides the figures for the eight most commonly mentioned drugs. The figures shown are unweighted.

**Table 5:** Multinomial regression of the social determinants of (definite) migraine and (definite) tension-type headache (n=2,526 women, n=2,280 men)^1^ Source: Study on headache, back and neck pain in Germany (2019/2020)

	Migraine (definite)			Tension-type headache (definite)		
	OR^2^	p-value	(95% CI)	OR^2^	p-value	(95% CI)
**Sex (Reference group: men)**						
Women	**4.68**	<0.001	(3.22–6.80)	**2.65**	<0.001	(1.85–3.80)
**Age group (Reference group: 18–<30 years)**						
30–<40 years	0.79	n.s.	(0.43–1.47)	1.65	n.s.	(0.84–3.25)
40–<50 years	**0.45**	<0.01	(0.26–0.80)	0.69	n.s.	(0.34–1.38)
50–<60 years	**0.22**	<0.001	(0.13–0.39)	**0.37**	<0.01	(0.20–0.66)
60–<70 years	**0.07**	<0.001	(0.03–0.13)	**0.20**	<0.001	(0.11–0.39)
≥70 years	**0.02**	<0.001	(0.01–0.04)	**0.07**	<0.001	(0.03–0.16)
**Education (Reference group: medium)**						
Low	0.49	n.s.	(0.25–0.99)	0.57	n.s.	(0.27–1.20)
High	0.95	n.s.	(0.70–1.29)	0.75	n.s.	(0.54–1.02)
**Unemployment** **(Reference group: employed/retired)**	2.11	n.s.	(0.76–5.83)	0.81	n.s.	(0.24–2.74)
**Partnership** **(Reference group: no partnership)**	1.05	n.s.	(0.73–1.52)	0.94	n.s.	(0.63–1.39)
**Social support (Reference group: medium)**						
Low	1.24	n.s.	(0.76–2.02)	1.03	n.s.	(0.55–1.92)
High	**0.63**	<0.05	(0.44–0.91)	0.77	n.s.	(0.54–1.12)

OR = odds ratio, 95% CI = 95% confidence interval, bold = significant (p <0.05), n.s. = not significant

^1^ All participants who answered ‘No’ to the initial question ‘Have you had a headache in the past twelve months?’ were used as the reference group in the multinomial logistic regression.

^2^ In multinominal logistic regression relative risk ratios (RRR) are computed to express the strength of ststistical associations. Usually, these are interpreted as odds ratios.

## Appendix

**Annex Table 1:** The sociodemographic characteristics of the study population compared with official statistics Source: Study on headache, back and neck pain in Germany (2019/2020), Destatis population update*

	Study on pain disorders	Official statistics*
**Sex**		
Women	52,6%	51,1%
Men	47,4%	48,9%
**Age group**		
18–29 years	9,2%	16,6%
30–39 years	11,4%	15,3%
40–49 years	13,8%	15,0%
50–59 years	21,6%	19,4%
60–69 years	21,7%	14,8%
≥70 years	22,4%	18,8%
**Education**		
Low	5,0%	17,5%
Medium (no Abitur)	29,7%	41,9%
Medium (has Abitur)	13,0%	15,4%
High	52,4%	25,2%

^*^ Official statistics based on the population update (as of 31 December 2018) and the results of the 2017 microcensus.
