# Corrigendum: Migraine and tension-type headache in Germany. Prevalence and disease severity from the BURDEN 2020 Burden of Disease Study

**DOI:** 10.25646/7683

**Published:** 2020-11-30

**Authors:** Michael Porst, Annelene Wengler, Janko Leddin, Hannelore Neuhauser, Zaza Katsarava, Elena von der Lippe, Aline Anton, Thomas Ziese, Alexander Rommel

Porst M, Wengler A, Leddin J, Neuhauser H, Katsarava Z et al. (2020)

Journal of Health Monitoring 5(S6): 2–24.

DOI 10.25646/6990

In the original version, the drug ‘metamizole sodium’ was shown twice in table 4, both as the active ingredient itself as well as a preparation containing the active ingredient (trade name ‘Novaminesulfone’).

In the corrected version, table 4 shows the order of medications after merging the two entries (i.e. assigning the brand ‘Novaminesulfone’ to the group of ‘metamizole sodium’). In the running text on page 14, the sentence “Therefore, triptans are particularly taken by people with migraine and are ranked at place four with 7.3%.” has been corrected accordingly. The correct sentence reads: “Therefore, triptans are particularly taken by people with migraine and are ranked at place five with 7.3%.”

The corrected version of the article is available at DOI 10.25646/6990.2

